# Supplementary material for: De Novo Transcription Responses Describe Host-Related Differentiation of Paracoccus marginatus (Hemiptera: Pseudococcidae)
Source: Insects. 2022 Sep 19;13(9):850. doi: 10.3390/insects13090850 (PMC9502998; doi:10.3390/insects13090850)
Supplement: Supplementary file 1 [file insects-13-00850-s001.zip › Supplementary Information (Figures S1-S7, Tables S1-S8).pdf]

Supplementary Information (Figures S1-S7, Tables S1-S8)

***De Novo* Transcription Responses Describe Host-Related Differentiation of *Paracoccus marginatus* (Hemiptera: Pseudococcidae)**

Li-Zhen Zheng[1], Jian-Yu Li[1], Meng-Zhu Shi[2], Yan-Ting Chen[1], Xiao-Yun He[2], Jian-Wei Fu[2*]

[1] Institute of Plant Protection, Fujian Academy of Agricultural Sciences, Fuzhou, Fujian, China,

[2] Institute of Quality Standards & Testing Technology for Agro-Products/Fujian Provincial Key Laboratory of Quality and Safety of Agricultural Products, Fujian Academy of Agricultural Sciences, Fuzhou, Fujian,China

[*]Corresponding author: fjw9238@163.com

Supplementary Information:

Figure S1. Modified plastic box and experimental breeding

Figure S2. Distribution of the length of all unigenes in *Pa. marginatus.*

Figure S3. GO enrichment analysis of the DEGs.

Figure S4. Scatterplot of enriched KEGG analysis of the up and down-regulated DEGs. (a) The down-regulated DEGs. (b) The up-regulated DEGs.

Figure S5. DEGs parted in the longevity regulation pathway-worm pathway of *Pa. marginatus.*

Figure S6. Correlation analysis between RT-qPCR and De novo data of 10 selected genes expression levels, respectively. Each color point represents a value of fold change of expression level at TF6 comparing with MF6.

Figure S7. DEGs parted in the Drug metabolism - cytochrome P450 pathway of *Pa. marginatus.*

Table S1. Primers used in qRT-PCR gene expression verification experiment.

Table S2. KOG_classification.

Table S3. Up and down regulated genes.

Table S4. KEGG_classification.

Table S5. UP-Pathway and DOWN-Pathway of DEGs.

Table S6. GO enrichment results of DEGs.

Table S7. KEGG pathway enrichment results of DEGs.

Table S8. DEGs related to digestive enzyme, detoxifying enzyme, reproduction and ribosome in *Pa. marginatus* transcriptome.

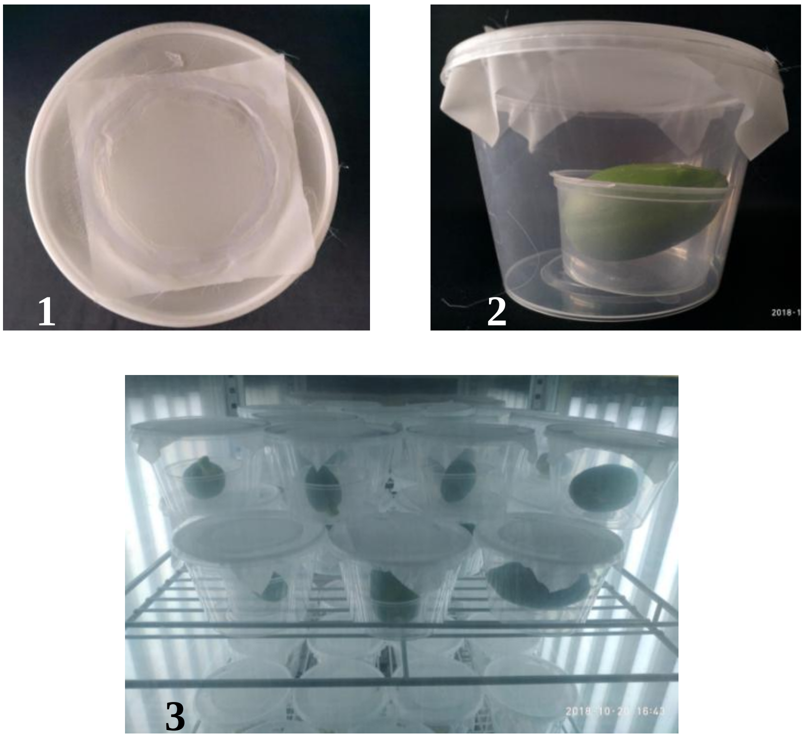

Figure S1. Modified plastic box and experimental breeding. 1 Reconstructed transparent plastic round box (top view)；

2. Reconstructed transparent plastic round box (front view)；

3. Cultivation of Paracoccus marginatus in artificial climate chamber.

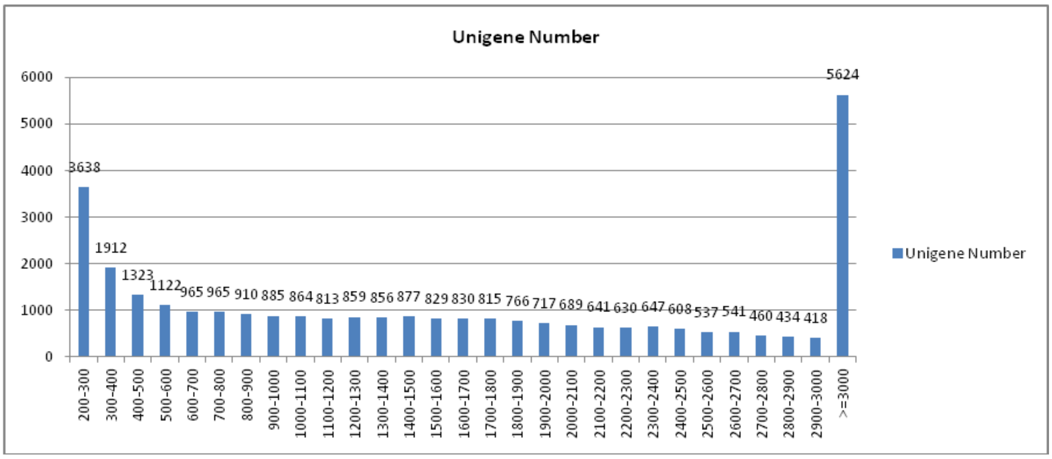

Figure S2. Distribution of the length of all unigenes in *Pa. marginatus.*

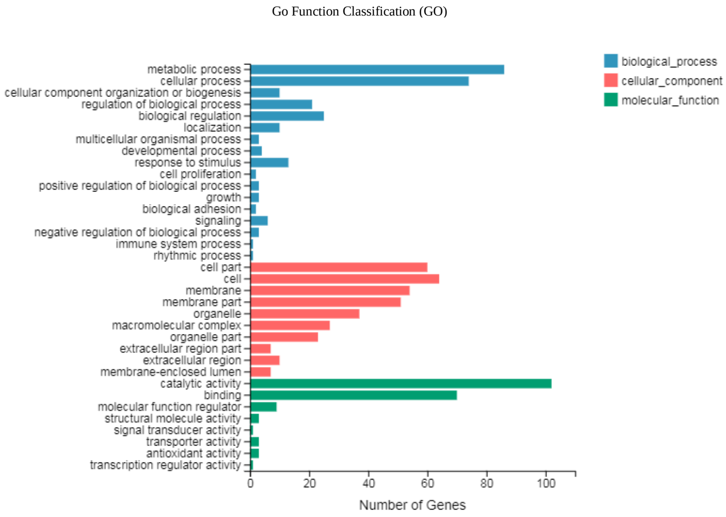

Figure S3. GO enrichment analysis of the DEGs.

(a)

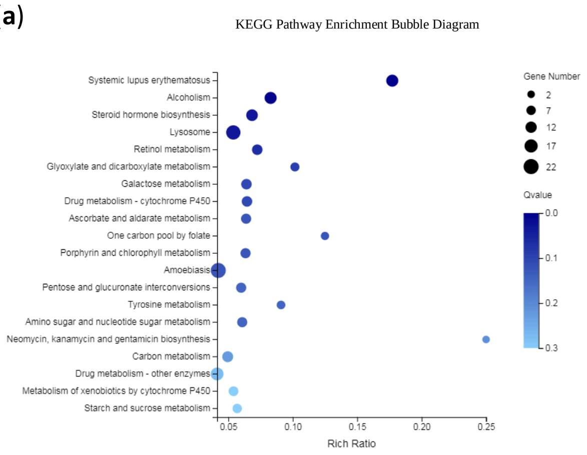

(b)

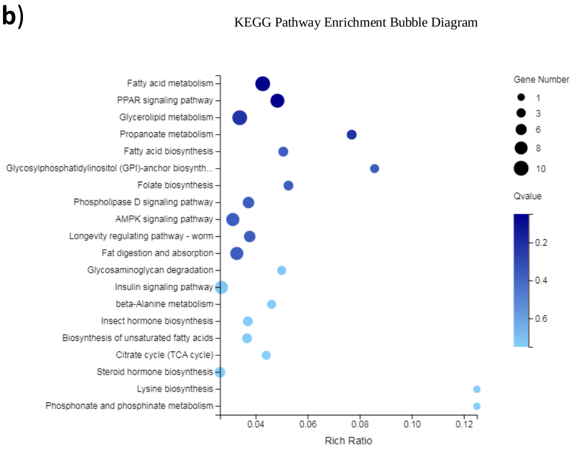

Figure S4. Scatterplot of enriched KEGG analysis of the up and down-regulated DEGs. (a) The down-regulated DEGs. (b) The up-regulated DEGs.

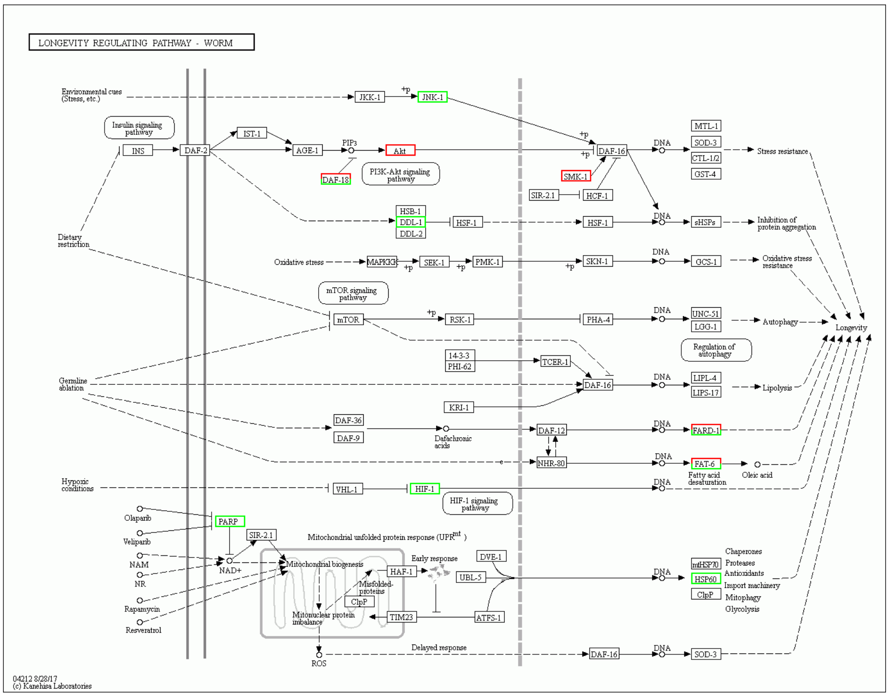

Figure S5. DEGs parted in the longevity regulation pathway-worm pathway of *Pa. marginatus.*

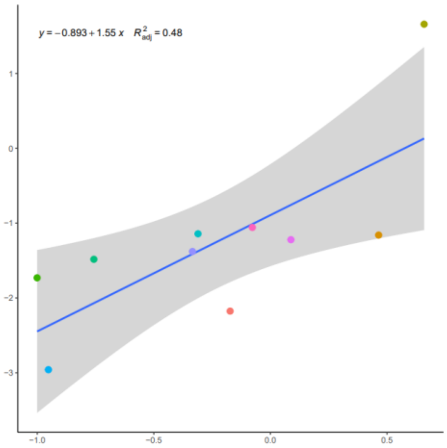

Figure S6. Correlation analysis between RT-qPCR and De novo data of 10 selected genes expression levels, respectively. Each color point represents a value of fold change of expression level at TF6 comparing with MF6.

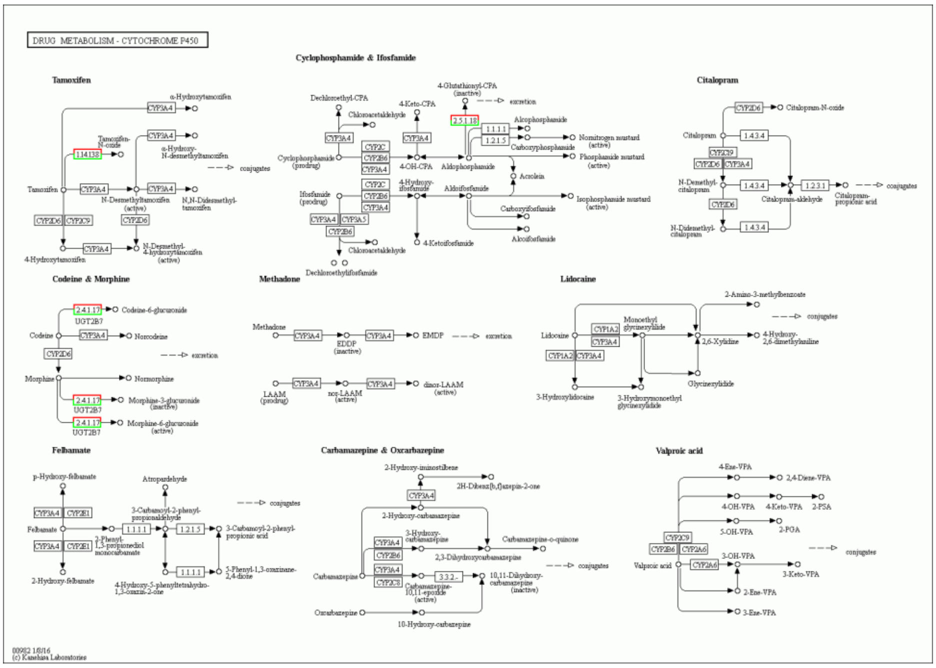

Figure S7. DEGs parted in the Drug metabolism - cytochrome P450 pathway of *Pa. marginatus.*
